# Historical Perspective and Medical Maladies of Alexander the Great

**DOI:** 10.7759/cureus.23925

**Published:** 2022-04-07

**Authors:** Shri K Mishra, Adam Mengestab, Shaweta Khosa

**Affiliations:** 1 Neurology, Olive View - University of California Los Angeles Medical Center, Los Angeles, USA; 2 Neurology, Keck School of Medicine of the University of Southern California, Los Angeles, USA; 3 Neurology, Scripps College, Claremont, USA

**Keywords:** history, cause of death, pancreatitis, encephalopathy, alexander the great

## Abstract

Alexander the Great (356 BC - 323 BC) was only 20 years old when he was named the next King of Macedonia after his father was assassinated. The following 11 years witnessed the evolution of an outstanding leader who expanded his empire from Egypt to the Indian frontier. Despite successfully conquering much of the world, he was afflicted with a febrile illness at the age of 32, which he battled for a mere 11 days before perishing. It has been almost 2,400 years since his death, but the exact cause remains a mystery. Did he die of natural causes or at the hands of conspirators? Numerous papers have been written about the illnesses suffered by Alexander, with the current evidence revealing a healthy 32-year-old man who developed fever and acute abdominal pain with rapid deterioration of his general condition leading to death within a short duration. We analyze various theories and discuss possible etiologies that may have contributed to his tragic death. Information was gathered from primary and secondary sources found through searching multiple online academic databases and the University of Southern California (USC), University of California Los Angeles (UCLA), and Harvard libraries. Unreliable sources and the unavailability of Alexander’s body for autopsy make reaching a definitive diagnosis an impossible task; however, based on existing information, we presume that he most probably died of a neurological cause due to acute necrotizing pancreatitis and encephalopathy secondary to peritonitis. Other potential causes include fulminant hepatic failure, acute demyelinating neuropathy or Guillain Barre Syndrome, and arsenic poisoning.

## Introduction and background

In July of 356 BC, in Pella, the ancient capital of Macedonia, King Philip II and one of his wives, Olympias, Princess of Epirote, were blessed with a male child they named Alexander. From birth, it was prophesied that he would go on to become one of the most powerful and successful commanders in history [1].

Aristotle, a pupil of Plato and the most famous philosopher of his time, was appointed to train 13-year-old Alexander in rhetoric, science, and philosophy. This training had a deep impact on the development of Alexander’s personality, helping him become a well-mannered, noble, and educated young man [2].

In 338 BC, Alexander’s father, King Phillip, married Eurydice, the niece of Attalus. At the wedding banquet, there was a violent altercation between Alexander and a drunken King Philip regarding the lawful successor of the kingdom, which led to Alexander being exiled from Macedonia alongside his birth mother Epirote [3]. After six months, the tension between the father and son had calmed and Alexander was able to return. In June 336 BC, King Philip was assassinated at a theater by Pausanias. Alexander, who was only 20 years old at the time, became the next King of Macedonia following his father’s death [2].

Macedonia was in poor shape when Alexander came to power. The neighboring Greek cities were so dissatisfied with the young king being named that they began revolting. Alexander marched to the Danube, overcame the opposition, and then marched towards Greece. Later, Alexander crushed the revolt of Thebes in 335 BC. This incident sent a clear message to the rest of the world about the consequences of disobeying Alexander. Subsequently, the Athenians apologized and became loyal to the Macedonians [2].

**Figure 1:**
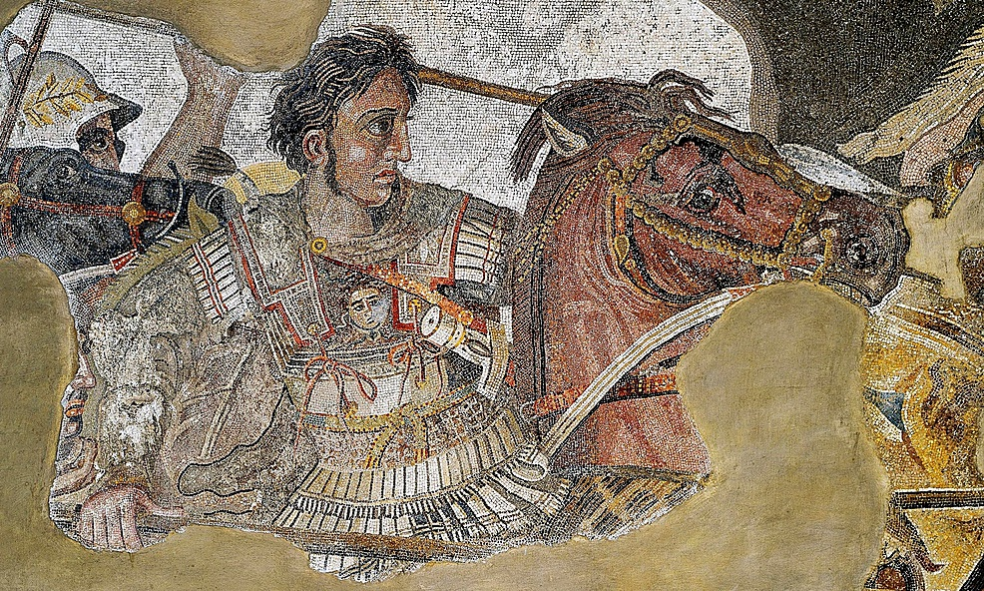
Alexander the Great, fighting the Persian king Darius III (Pompei mosaic, from a 3rd century BC original, now lost) Public Domain

Right from the start, Alexander had a hunger for more power. His series of successful military campaigns lasted the duration of his reign, amassing conquests from Gibraltar to Punjab. He invaded Persia the following year in 334 BC, beating Darius III in two decisive battles (Figure 1). With victories in the cities of Granicus and Issus in modern-day Turkey, Alexander had conquered the Persian Empire [3]. Next, he continued taking cities along the Mediterranean with siege victories in Tyre and Gaza en route to Egypt, which had been under Persian rule at the time. Finally, Alexander crowned himself Pharaoh and founded Alexandria in 331 BC on the North Coast along the River Nile. Shortly afterward, in the city of Gaugamela in modern-day Iraq, Alexander would again face Darius III, who had now amassed an army twice the size of Alexander’s. Again, Alexander would take a bold victory, forcing Darius to flee and abandon his army. Darius III was then killed by his own troops [2].

It is believed that Limnus, a Macedonian, had plans to kill Alexander by hiring Nicomachus, but Nicomachus refused to participate. Instead, Nicomachus revealed Limnus’s plan to his brother, and both the brothers went to Philotas, son of Parmenio, who was second in command of Alexander’s army and a loyal friend of Alexander’s father [2]. The brothers tried to meet Alexander, but Philotas kept avoiding them. The brothers were finally able to meet Alexander with the assistance of a third person and told him the entire story. Alexander wanted to examine all the facts, but before he could, his soldiers reported that Limnus had killed himself prior to his arrest. Alexander grew suspicious and executed both Philotas and his father, Parmenio. Soon after this incident, during a party, Alexander had a heated argument with Cleitus, one of his close friends. On one occasion, Callisthenes was invited to speak about the Macedonians’ wrong deeds to learn from them. Although Callisthenes was a powerful speaker, he had poor judgment. He said something offensive, which made Alexander furious. Some say that Alexander executed Callisthenes while others believe he died in prison [3].

Alexander launched a final campaign in India in 327 BC. He found an ally in King Taxiles, who offered Alexander the use of his city of Taxila and any supplies needed if he could defeat King Porus [3]. In 326 BC, Alexander defeated Porus’ army in a fierce battle on the Hydaspes River; Alexander had been victorious over every army from Greece to India. Nevertheless, in doing so, he lost thousands of his men and his favorite horse Bucephalus. Alexander’s men, at this point, had no enthusiasm to continue on the warpath across the Ganges; they were depleted of energy and reluctant to fight [4]. Seeing the state of his troops, Alexander finally decided to return.

It took many weeks to cross the Indus River and almost another week to cross the Gedrosian desert. It was a painful experience, as thousands of his soldiers died in the desert. Out of the 120,000 infantry and 15,000 cavalries that Alexander took into India, only one in four returned [4]. Furthermore, the invasion into India was a nightmare, as Alexander was hit with an arrow while taking over the Mallians, and several soldiers died in the Gedrosian desert. After hearing this news, many people started revolting against Macedonian rule, but Alexander was not disappointed and wanted to go on a new mission. This time, he proposed to sail around Africa to the Pillars of Hercules in Gibraltar [4].

## Review

Examination of the leading theories behind Alexander’s mysterious death

**Figure 2:**
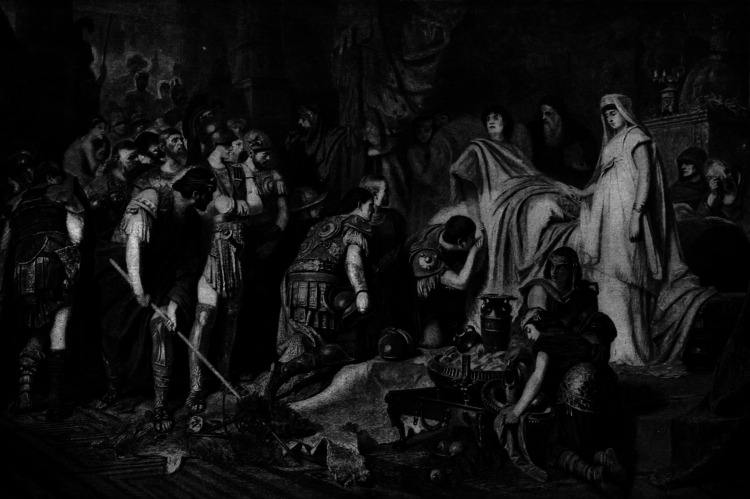
Death of Alexander the Great by Karl T. von Piloty (1826-1886) Public Domain

Alexander died on June 11, 323 BC, in the late afternoon at the palace of Nebuchadnezzar II in Babylon (Figure 2). The text of historians like Plutarch and Arrian (1st and 2nd century AD) is based on the contents of the ‘Royal Journal,’ a diary maintained by the court of Alexander the Great [5]. The possible causes of his mysterious death are thoroughly listed below and grouped into five categories: alcohol-induced, infectious disease, poisoning, internal organ damage, and other complications.

Alcohol-Induced

Acute necrotizing pancreatitis with encephalopathy:* *Heavy alcohol intake can cause acute pancreatitis. Epigastric pain is the primary symptom that varies from mild to severe and is usually constant. It usually worsens when the patient is lying on his back with radiation to the flanks, back, or both. The patient usually sits in bed leaning slightly forward to reduce the pain and discomfort. Nausea and vomiting are present in more than two-thirds of patients. It can lead to sepsis, which may lead to death. However, Alexander had transient, rather than constant, abdominal pain. His fluctuating fever increased over approximately 11 days, which is not characteristic of septic necrotizing pancreatitis, which usually has a very acute and rapid deterioration, quickly leading to death [6]. The ascending weakness could possibly be due to Guillain Barre Syndrome, which is seen in some cases of acute pancreatitis [7].

Delirium tremens: Alexander periodically consumed large quantities of alcohol in the form of undiluted wine. During his illness, it is said that he opted to drink wine rather than water to quench his thirst [2]. Based on the circumstances, this increased intake could be interpreted as the cause of Alexander’s demise; however, the most notable of Alexander’s clinical symptoms, his fever, cannot be explained by excessive alcohol intoxication or withdrawal. Furthermore, vomiting, which is commonly observed in cases of delirium tremens, was absent. There was also no record of visual or tactile hallucinations. Therefore, Alexander did not meet the Diagnostic and Statistical Manual of Mental Disorders, Fifth Edition (DSM-5) criteria for alcohol use disorder nor did he have the DSM-5 outlined symptoms for alcohol withdrawal and delirium tremens [8].

Alcoholic cirrhosis: Although Alexander’s aforementioned penchant for drinking wine was well-documented, there is no mention of cirrhosis symptoms in his records as ascites, jaundice, and edema were not observed [2]. As noted above, it was believed that Alexander drank wine during his illness only to quench his thirst.

Infectious Disease

West Nile encephalitis: According to the John Marr and Charles Calisher theory, Alexander’s death is attributed to complications of West Nile infection [9]. This theory is based on Plutarch’s description of the death of a flock of ravens as Alexander entered Babylon. West Nile virus was identified in Israel in 2000 and in America in 1999. Birds are the amplifying hosts. Diseased birds manifest various symptoms, including abnormal head and neck postures, ataxia, tremors, circling, disorientation, and impaired vision [9]. Mortality rates in infected birds are very high. West Nile encephalitis in humans usually manifests with mild fever, headache, body aches, and skin rash. High-grade fever, disorientation, convulsions, muscle weakness, paralysis, and coma occur in only a small percentage of severe cases, and death is rare. Acute flaccid paralysis as a complication of West Nile virus was noted in the United States in 1999 and later. In most cases, fever usually does not increase or last more than two weeks. Mental confusion and muscle weakness are the initial symptoms, but Alexander was well-oriented and performed his daily routine in the initial phase of his illness. Only later, during the course of his illness, he developed delirium and was unable to speak, shortly after which he died [2]. Encephalitis itself became a more frequent complication of West Nile virus fever in 1996, suggesting the recent appearance of a more pathogenic viral strain. However, serious neurological complications of West Nile virus only occur in less than 1% of patients infected [9].

Typhoid fever: David Oldach was the first to propose that Alexander died of typhoid based on the symptoms described by Plutarch [10]. It is suspected that Alexander may have been the victim of a poorly treated or untreated case of typhoid fever, complicated by enteric perforation. This could explain the sudden onset of a single episode of sharp abdominal pain. Furthermore, instances of typhoid fever complicated by intestinal perforation with subsequent peritonitis have been described in prior literature and can lead to acute demyelinating neuropathy or Guillain Barre Syndrome [7]. In typhoid fever, patients may present with delirium and splenomegaly in the end-stage of the disease. Patients with peritonitis can present with clammy perspiration, a fast, thready, and weak pulse, and Hippocratic facies. Due to the lack of antibiotics, the course was almost invariably fatal during Alexander’s period. Alexander’s face in his final days was described as expressionless; although bedridden, he was responsive enough for final farewells from his troops. The apathetic facies and delirium known as typhoid state that can accompany typhoid fever fit this description [11]. However, Oldach said that he and others believe that Alexander’s body’s lack of decay after death is a legend created by those who survived him, likely a red herring in his case, and should be discarded [10]. Alexander’s immune system may have been weakened after he returned from India due to the severe stress that he was under. Typhoid fever is a bacterial infection caused by Salmonella typhi and spreads by the fecal-oral route. Without antibiotics or other modern medical therapies, it is often fatal. Risk exists to travelers visiting countries where it is endemic; it was present in Babylon at the time [11]. Alexander traveled from Greece to the Indian frontier, which had many endemic areas of Salmonella typhi. The typical course of typhoid fever includes fever, loss of appetite, weakness, headache, constipation, and a typical, gradual, stepwise increase in severity of fever during the first week. There is a gradual increase in fever in the second week, and the skin becomes dry and hot. In the third week, fever continues to rise, and complications such as delirium, stupor, intestinal perforation, peritonitis, and intestinal bleeding can occur. The main issue with the validity of this theory is the timing of symptoms, as intestinal perforation in patients with typhoid fever often occurs in the third week or later.

Malaria: Alexander the Great bathed in the Euphrates River, which was infested by mosquitoes that carry malaria. Some believe that he became sick due to a relapse of malaria he had contracted in 336 BC. Malaria is caused by Plasmodium and transmitted by female Anopheles mosquitoes. He traveled extensively in malaria-endemic areas, especially in the final years of his life [12]. The symptoms and signs include chills, headache, muscle ache, and fever without periodicity in the first week. During the second week, chills followed by fever and sweating, a typical malarial paroxysm, are observed. After that, periodicity develops with intermittent or remittent fever. In between the episodes, the patient is afebrile and is largely asymptomatic. The third week shows a gradual decrease of malarial paroxysm, including the periodic fever. Patients typically improve to baseline after this acute/subacute period. There are multiple factors supporting this theory. Some symptoms seen in cerebral malaria, such as fever, chills, sweating, prostration, muscle ache, progressive weakness, stupor, decreased sensorium, and delirium, which can be seen with Plasmodium (P.) falciparum, were present in Alexander’s condition [11]. Although acute abdominal pain is not a typical feature of P. falciparum infection, it can occur due to intestinal ischemia secondary to mesenteric arterial thrombosis, a complication known as Algid malaria. Therefore, cerebral malaria might cause progressive neurological deterioration. Points against this theory are the absence of dark urine usually seen in P. falciparum, the absence of intermittent fever, the apathetic faces, and clinically, abdominal pain is more common in typhoid fever. Additionally, today, most malaria in Iraq/present-day Babylon is due to P. vivax, in which abdominal pain is absent [12].

Influenza: There was no report of any death in Alexander’s camp with similar signs and symptoms, so the chance of an isolated influenza case as a cause of his death is very remote.

Poisoning

Other theories of poisoning: Some believe he was poisoned. Various theories about who may have poisoned him include unsatisfied lieutenants, his jealous wife Roxane, his Regent of Macedonia Antipater, or his teacher Aristotle [5]. Some commonly known poisons from ancient times that induced fever were ergot, mycotoxins, and alkaloids [13]. However, mycotoxins are unlikely as they do not cause a sustained high fever.

Strychnine poisoning: Graham Phillips’s theory is that Alexander’s wife, Roxane, poisoned him with a little-known toxin of that period, which was extracted from the strychnine plant [14]. Strychnine grew only in the Indus Valley, so Roxane, being from Bactria located in present-day Afghanistan, could have had knowledge of and access to it. Its taste can be easily masked with wine. Signs and symptoms typically include violent seizures, muscle stiffness, tachycardia, tachypnea, apnea, and death. However, strychnine causes convulsions and muscle rigidity, both of which were absent in Alexander’s record. A gradual rise in fever and delirium are atypical of strychnine poisoning.

Toxicity of arsenical compounds: Plutarch mentions that Aristotle procured arsenic to poison Alexander. Arsenical compounds were used to treat ulcers and syphilis. In toxic doses, extensive transmural inflammation of enteric mucosa, severe abdominal pain, hemorrhagic gastroenteritis, hepatic necrosis [15], pulmonary edema, hypotension, and shock may occur. If these events are not fatal, progressive neuropathy, which may be indistinguishable from Guillain-Barre Syndrome, may yet claim the patient’s life [7]. However, arsenical compounds usually do not cause a sustained high fever.

Acute lead poisoning:* *Lead water pipes and lead-based pottery glazes used as storage for homemade wine were responsible for epidemics of lead poisoning in ancient Rome [3]. Acute poisoning can lead to severe colicky abdominal pain, fatigue, paralysis, and encephalopathy, but again, acute lead poisoning does not explain the pattern of Alexander’s fever.

Belladonna poisoning: Belladonna poisoning may produce vocal cord paralysis, which could explain why Alexander, although conscious, could not speak in his last days. However, this does not explain the nature of Alexander’s fever.

Methanol poisoning: Methanol poisoning can produce peripheral neuropathy, which can lead to generalized weakness. However, visual symptoms and vomiting, typical of methanol poisoning, are not mentioned in Alexander’s records.

Adverse drug reaction (white hellebore): Another theory is the possibility of repeated poisoning with white hellebore [14]. The drug possesses strong purgative and anti-helminthic properties but is violently narcotic and causes bradycardia. However, it does not explain the sudden onset of acute abdominal pain and the fever pattern seen in Alexander’s case.

Internal Organ Damage

Amoebic liver abscess rupture: Although the parasitic amoebic infection was present at that time, a vast majority of cases are sub-acute. Symptoms typically develop gradually over weeks to months. Initial symptoms are non-specific, but in later stages, right upper quadrant pain becomes the main symptom. Fever is usually present but is intermittent and rarely exceeds 40 degrees Celsius. The patient is chronically ill with fever and abdominal tenderness in the right upper quadrant. Rupture of an amoebic abscess can lead to sudden severe abdominal pain. However, the time period and clinical features between the rupture of an abscess, presumably into the peritoneal cavity, and Alexander’s subsequent signs and symptoms point against a ruptured amoebic abscess as the cause of his death [8].

Perforated peptic ulcer (duodenal/gastric): Prior to the 20th century, it was a disease of young males that caused acute abdominal pain. However, most patients have a history of indigestion prior to perforation. Therefore, the nature of Alexander’s fever cannot be explained by this theory.

Right lower lobe (RLL) pneumonia, recrudescent empyema, and pleurisy: Acute right upper quadrant pain can be produced by irritation of diaphragmatic pleura. Alexander had a history of acute pneumonia in 333 BC and suffered a hemopneumothorax one year earlier due to an open chest wound caused by an arrow [2]. However, the abrupt onset and severity of pain and the course of his fever and illness are more suggestive of typhoid fever complicated by bowel perforation.

Acute cholecystitis/acute ascending cholangitis: Cases of cholecystitis are commonly characterized by persistent abdominal pain in the right upper quadrant. While cases of cholangitis typically present with right upper quadrant pain, fevers, and jaundice, collectively comprising what is known as Charcot’s triad. Sepsis and delirium have also been observed in cholangitis cases, which when presenting alongside Charcot’s triad, comprise Reynold’s pentad. Obstruction of the biliary tree can occur due to stones or parasitic infection and is less likely due to tumors in patients younger than 32 years old. However, gall bladder perforation is rare at age 32, and the absence of jaundice in the records argues against this theory.

Other Complications

Carotid dissection: Andrew Williams and Robert Arnott suggest traumatic dissection of one of his internal carotid arteries six years prior to his death, in 329 BC in the Persian city of Cyropolis, caused by being hit by a stone from a local slinger, which struck Alexander’s head/neck and led to transient blindness and loss of speech [16]. Signs and symptoms usually include mild headache, Horner’s syndrome with unilateral dilatation of the pupil, cranial nerve palsies, transient ischaemic attack (TIA), aphasia, loss of consciousness, hemiparesis, occasional ischemic optic neuropathy, transient unilateral blurring, or loss of vision. The patient may be initially asymptomatic but can quickly develop symptoms due to embolization of thrombus at the site of dissection after an interval of a few hours to days. However, this theory cannot explain the course of his fever and illness. In addition, there is a lack of cardinal signs and symptoms in the long period between dissection and death, such as hemiparesis, hemisensory loss, headache, and Horner syndrome.

Complications of the congenital scoliotic syndrome: Hutan Ashrafian proposed this theory based on numismatic and sculpture studies [17]. He noticed a portrait of Alexander on a Greek coin revealing a facial horn which suggests a possibility of scoliotic epidermal nevus syndrome [18]. Additionally, adults with Klippel-Feil syndrome may present with a short neck, cervical deformity, oculomotor findings, facial asymmetry, abnormal gait, orthodontic defects, and a family history of limb irregularities. Neurofibromatosis can result in malaise, pyrexia, and neurological deterioration. Epidermal nevus syndrome could account for cervical scoliosis, familial musculoskeletal deformities with seizures, ocular irregularities, and facial horns. Repeated cervical trauma can cause quadriplegia. Furthermore, the asymmetry of his head, shoulders, and neck axis and asymmetry of the eyes with limited adduction in the marble bust of 2nd and 1st BC may indicate the exotropic Duane syndrome II. Heterochromia irides and abnormal dentition may be associated with it. However, it is difficult to establish the diagnosis in the absence of Alexander’s body. The use of iconographic records, such as numismatics and sculpture, to provide evidence of clinical symptoms is shown to be highly misleading. However, there is a whole series of extant sculptures of Apollo, young athletes, women poets, such as Sapho, prior to or contemporary with the bust of Alexander, which display similar features as enumerated by Ashrafian as being abnormal [17]. 

## Conclusions

As detailed above, Alexander the Great was the greatest warrior of his time. He died in the late spring of 323 BC in Babylon, and the precise cause of his death has never been ascertained. It is believed that he had many past and present illnesses, including post-traumatic transient cortical blindness, malaria, toxin poisoning, and alcoholism, which may have contributed to his death. The exact cause of his death is difficult to establish. Based on the literature review, the chain of events leading to his terminal illness seems to highlight undue stress from several sources, including demoralization due to his lack of command over his soldiers, which may have weakened his immune system, making him more susceptible to illness. He had been drinking very heavily for a few days before the onset of his illness. He suffered from acute abdominal pain followed by febrile illness, delirium, dysarthria leading to coma, and death. In addition, we feel Alexander suffered from acute necrotizing pancreatitis complicated by peritonitis leading to disseminated cerebritis as well as bacterial meningitis causing encephalopathy; this ultimately resulted in coma and his subsequent death. Other possible causes include Guillain Barre syndrome and poisoning from arsenic compounds. Unless Alexander the Great’s remains are recovered, we may never be able to determine the exact cause of his death with certainty.
